# Whole-Genome Analysis of *Staphylococcus aureus* Isolates from Ready-to-Eat Food in Russia

**DOI:** 10.3390/foods11172574

**Published:** 2022-08-25

**Authors:** Yulia Mikhaylova, Andrey Shelenkov, Aleksey Chernyshkov, Marina Tyumentseva, Stepan Saenko, Anna Egorova, Igor Manzeniuk, Vasiliy Akimkin

**Affiliations:** Central Research Institute of Epidemiology, Novogireevskaya str., 3a, 111123 Moscow, Russia

**Keywords:** foodborne pathogen, antibiotic resistance, virulence determinants, multidrug resistance, pathogenic potential, CRISPR/Cas system, genomic epidemiology

## Abstract

This study provides a thorough investigation of a diverse set of antimicrobial resistant (AMR) *Staphylococcus aureus* isolates collected from a broad range of ready-to-eat (RTE) food in various geographic regions of Russia ranging from Pskov to Kamchatka. Thirty-five isolates were characterized using the whole genome sequencing (WGS) analysis in terms of clonal structure, the presence of resistance and virulence determinants, as well as plasmid replicon sequences and CRISPR/Cas systems. To the best of our knowledge, this is the first WGS-based surveillance of Russian RTE food-associated *S. aureus* isolates. The isolates belonged to fifteen different multilocus sequence typing (MLST)-based types with a predominant being the ones of clonal complex (CC) 22. The isolates studied can pose a threat to public health since about 40% of the isolates carried at least one enterotoxin gene, and 70% of methicillin-resistant (MRSA) isolates carried a *tsst1* gene encoding a toxin that may cause severe acute disease. In addition, plasmid analysis revealed some important characteristics, e.g., Rep5 and Rep20 plasmid replicons were a “signature” of MRSA CC22. By analyzing the isolates belonging to the same/single strain based on cgMLST analysis, we were able to identify the differences in their accessory genomes marking their dynamics and plasticity. This data is very important since *S. aureus* isolates studied and RTE food, in general, represent an important route of transmission and dissemination of multiple pathogenic determinants. We believe that the results obtained will facilitate performing epidemiological surveillance and developing protection measures against this important pathogen in community settings.

## 1. Introduction

The urgency of the food safety issue as one of the main risk factors for public health and gene pool maintenance increases every year [1]. The vast majority of foodborne outbreaks caused by antimicrobial-resistant pathogens are the result of the consumption of contaminated foods of either animal origin or multi-ingredient foods [2]. Foodborne illnesses are considered a major source of morbidity and mortality, mainly in such susceptible groups as infants, the elderly, and immunocompromised people. In healthy adults, foodborne infections and intoxications are usually mild and self-limiting [3].

*Staphylococcus aureus* is a versatile opportunistic pathogen, which can survive in diverse environments, grow in many types of foods, and cause food poisoning [2]. The wide application of molecular genotyping techniques revealed that *S. aureus* is a clonal bacterium with a limited number of genetic lineages, or clonal complexes (CCs), being predominant in the staphylococcal population worldwide [4]. This species represents a serious threat to human health and constitute one of the main challenges to the food industry [5]. In particular, staphylococcal intoxications are ranked the third in the world among all foodborne infections in terms of occurrence frequency [6,7]. This bacterium has an extraordinary capacity for acquiring new antimicrobial resistance characteristics. Thus, the problem of food intoxication became more complicated due to the presence of *S. aureus* isolates harboring a broad spectrum of antibiotic resistance and virulence determinants. Moreover, in such cases, ready-to-eat (RTE) food, which does not require thermal processing before consumption, represents a vehicle for spreading antibiotic-resistant microorganisms [8].

The presence of antibiotic-resistant staphylococci in RTE food usually is not investigated, especially by whole genome analysis, and only the data from a small number of studies are currently available. In most studies, the detection of molecular characteristics of antimicrobial resistance, virulence, and genotypes, as well as typing of the food strains, were performed by PFGE and PCR analyses [8,9,10,11,12]. Detection of methicillin-resistant *S. aureus* (MRSA) strains in Chinese food products has been reported for a variety of foods such as raw meat, rice flour, vegetable salads, sandwiches, meat products, and eggs [13]. This study showed the geographical variation of *S. aureus* isolates from sushi sold in Beijing and Copenhagen. In addition, a large-scale microarray analysis describing the genome composition of 267 *S. aureus* isolates sampled from 244 RTE-foods in Switzerland was performed, which showed the pathogenic potential of the isolates studied [14]. The results revealed that one-third of the tested isolates had at least one major enterotoxin gene (*sea*-*see*), and the toxic shock syndrome gene (*tsst*), while various genes associated with antimicrobial resistance, including genes involved in resistance to beta-lactams (*blaZ*), methicillin (*mecA*), and vancomycin (*vanB*), were also detected. These *S. aureus* strains mostly belonged to clonal complexes commonly detected in humans colonized or infected with *S. aureus* (CC8, 15, 30, and 45).

Whole genome analysis was applied in a previous study in China examining the prevalence and characterization of antimicrobial-resistant *S. aureus* in sushi and pork from a large number of outlets in Beijing [15]. Studying a large heterogeneous data set of *S. aureus* isolates collected from food and from individuals from many provinces of China over a nine-year period showed that Chinese and European strains of MRSA have evolved differently [16].

There is a lack of information available on the epidemiology and whole genome characterization of *S. aureus* sampled from RTE food in Russia. In this paper, we described for the first time, to the best of our knowledge, the variation in lineages, antimicrobial resistance (AMR) and virulence genes, plasmid sequences, and CRISPR/Cas system carriage for *S. aureus* isolates from RTE food collected in various geographic regions of Russia using whole genome sequencing (WGS) analysis. The main goals of investigations were to obtain epidemiologically related data, such as to perform isolate typing using various molecular classification schemes, as well as to reveal the presence of antibiotic resistance genes, virulence factors, plasmids, and CRISPR/Cas systems in the genomes of the isolates studied.

## 2. Materials and Methods

### 2.1. Determination of Antibiotic Susceptibility

**Figure 1:**
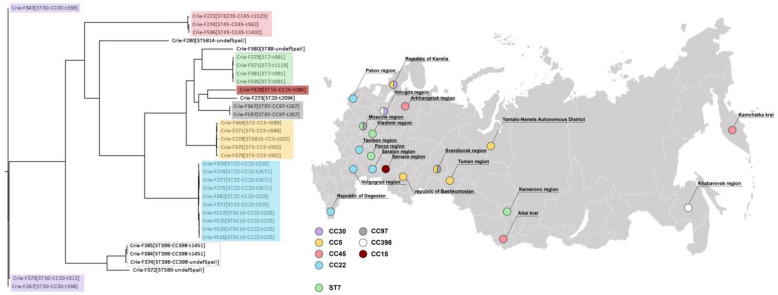
Typing, geography, and phylogenetic analysis of *S. aureus* isolates from ready-to-eat (RTE) food studied.

All isolates were identified down to a species level by time-of-flight mass spectrometry (MALDI-TOF MS) using the VITEK MS system (bioMerieux, Marcy-l’Étoile, France). The susceptibility was determined by the disc diffusion method with the Mueller–Hinton medium (bioMerieux, Marcy-l’Étoile, France) and disks with antibiotics (BioRad, MarneslaCoquette, France), as well as by the boundary concentration method on VITEK2 Compact 30 analyzer (bioMerieux, Marcy-l’Étoile, France). The isolates were tested for susceptibility/resistance to the following groups of antimicrobial drugs: aminoglycosides, beta-lactams, fluoroquinolones, glycopeptides, lincosamides, penicillins, and tetracyclines. Specific antibiotics are presented in Figure 1, including additional antimicrobial compounds, such as fosfomycin, fusidic acid, trimethoprim-sulfamethoxazole, and linezolid. The panel of antimicrobial compounds included for testing in this study was chosen according to the EUCAST and CLSI recommendations. To interpret the results obtained, the EUCAST clinical breakpoints, version 11.0 was used https://www.eucast.org/clinical_breakpoints/ (accessed on 20 April 2022) where available.

### 2.2. Sample Collection, DNA Isolation, Sequencing, and Genome Assembly

Thirty-five isolates were obtained from different types of ready-to-eat food during the period of 2019–2020 (Table S1). Sample collection was carried out in cafes and restaurants by eight Russian Federal Centers of Hygiene and Epidemiology located in Moscow, Saint-Petersburg, Nizhny Novgorod, Ekaterinburg, Rostov-on-Don, Stavropol, Novosibirsk and Khabarovsk for the purposes of food poisoning monitoring and epidemiological surveillance. The majority of the samples were isolated from the meat and chicken products (11 isolates), ten isolates were collected from salads, and six samples were isolated from fish products including rolls, while the remaining eight samples were obtained from potatoes, porridge, soup, yogurt, omelets, and bread (Table S1). All the isolates mentioned above were selected for WGS based on their resistance to two or more antimicrobial compounds from different groups.

Genomic DNA was isolated with DNeasy Blood and Tissue kit (Qiagen, Hilden, Germany) and used for paired-end library preparation with Nextera™ DNA Sample Prep Kit(Illumina^®^, San Diego, CA, USA) and WGS of all isolates on Illumina^®^ Hiseq platform (Illumina^®^, San Diego, CA, USA). Assemblies were obtained using SPAdes version 3.11 and 3.12 [17]. Genome sequences were uploaded to Genbank under the project number PRJNA823522.

### 2.3. Data Processing

Assembled genomes were processed using a custom software pipeline including a set of scripts for seamless integration of various available software tools [18,19]. Resfinder 4.0 database was used for antimicrobial gene identification (https://cge.cbs.dtu.dk/services/ResFinder/, accessed on 20 April 2022) and VFDB (http://www.mgc.ac.cn/VFs/main.htm, accessed on 20 April 2022) for virulence factor determination. CRISPRCasFinder [20] was used to identify the presence of CRISPR/Cas systems and spacers in the genomes studied.

The methods of multilocus sequencing typing (MLST) and Spa-typing, which are commonly used to study the global epidemiology and population structure of *S. aureus*, were applied to isolate typing. Agr (Accessory gene regulator) typing was also performed for our *S. aureus* isolates. This method involves the determination of variable regions from the cluster consisting of five genes (*agrABCD* and d-hemolysin). Four groups of agr (I–IV) are defined based on the types of variable regions [21].

The analysis of cgMLST schemes was performed using MentaList software (https://github.com/WGS-TB/MentaLiST, version 0.2.4), and the tree was built using PHYLOViz online (http://online.phyloviz.net, accessed on 20 April 2022).

## 3. Results

### 3.1. Isolate Typing

The results of the isolate typing using MLST and Spa-types are presented in Table S1 and in Figure 1. The isolates belonged to fifteen different MLST-based sequence types, while a major part of them belonged to seven clonal complexes (CCs). The dominant lineage in our collection was CC22 comprising ten isolates from five geographic regions of the European part of Russia. The CC22 isolates from the Pskov region differed by ST (6110), and the Dagestan isolates of the same lineage had a distinct SpaII type (t2571). The majority of CC22 isolates were characterized by the same SpaII (t223), and all members of this clonal group in our collection belonged to AgrI type.

The isolates of CC5 were collected from distant regions. The samples from the Tumen region and Yamal-Nenets autonomous district were characterized by the same typing pattern, while the isolate from the Sverdlovsk region differed from the Karelian sample by ST, and the Bashkir isolate had a distinct SpaII type.

Four isolates collected in Moscow, Penza, Vladimir, and Kemerovo regions belonged to ST7. The latter region is located quite far from the first three but was characterized by the same typing patterns as the isolates from Penza and Vladimir. The sample from Moscow differed by SpaII type.

The lineage CC45 was presented by three isolates collected from the Far Eastern part of Russia (Altai, Khabarovsk, and Kamchatka) which differed from each other by SpaII types. The clonal groups CC30 and CC398 included three samples, which also had different SpaII types. It is worth noting that two isolates of these clonal complexes were collected from the Vologda region. Two isolates of CC97 obtained from the Moscow and Sverdlovsk regions had the same typing patterns.

The results of Spa-typing of four isolates (Crie-F280, 372, 374, and 380) collected from different regions and belonging to different STs (5814, 580, 398, and 88, respectively) were particularly interesting. They were characterized by unique four respective sequences of short Spa-gene repeats, which were not presented in spaserver.ridom.de, indicating novel Spa-types (Table S1).

Typing analysis of the samples studied did not show a correlation between the typing patterns and the geographical origin of the isolates (Figure 1). Antimicrobial-resistant *S. aureus* isolates of RTE food origin of some clonal lineages (CC5, CC97, and ST7) were collected from very distant geographic regions of Russia, while CC30 and CC22 were observed in the European part only (Table S1 and Figure 1).

More detailed typing that allows better isolate discrimination can be achieved by cgMLST profiling, which involves a comparison of 1861 genes common to the most existing isolates (https://www.cgmlst.org/ncs/schema/schema/141106/, accessed on 20 April 2022). The minimum spanning tree for the isolates is presented in Figure S1, and the complete profiles are listed in Table S2. The threshold for the number of allele differences sufficient for assigning a particular isolate to a single strain can be determined ad hoc, but usually, the value of 15 is used for *S. aureus* [22]. Four ST6110 isolates from the Pskov region obtained from rolls differed from each other by the only allele of the cgMLST profile and only in Crie-F524/Crie-F527 pair. Three ST22 and two ST398 isolates from Dagestan and Khabarovsk regions, respectively, did not have any differences in cgMLST profiles, while two Karelian ST30 isolates (Crie-F267 and Crie-F343) differed by three cgMLST alleles. The latter two isolates were considered to belong to a single strain according to the threshold defined for *S. aureus* [22]. It was interesting that the samples from Dagestan and the Republic of Karelia were collected from different types of RTE food (bread, soup, salad, and trout caviar). Such a variety of sources may indicate the contamination with *S. aureus* of kitchen facilities located in the cafés/restaurants producing this RTE food.

### 3.2. Antimicrobial Resistance Phenotypes and Genotypes

**Figure 2:**
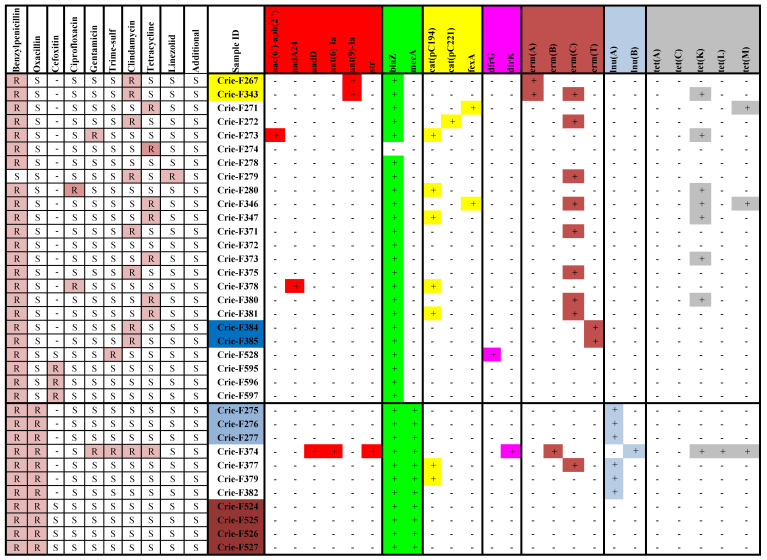
Phenotypic and genomic antimicrobial resistance profiles of *S. aureus* isolates from ready-to-eat food. ‘Trime-sulf’ represents trimethoprim-sulfamethoxazole. ‘Additional’ antibiotics, to which all isolates were susceptible, include fosfomycin, fusidic acid, and tigecycline. Antibiotics belonging to the same group are colored with the same color. ‘+’ means that the gene is present in the isolate, ‘-‘ – that the gene was not found. ‘R’ means that a given isolate was resistant to particular antibiotic, ‘S’ – that an isolate was susceptible to this drug. The isolates found to belong to the same strain are highlighted with color.

The results of antibiotic susceptibility testing of the isolates included in the study and their antimicrobial resistance gene profiles are presented in Figure 2. All the isolates except Crie-F279 were susceptible to benzylpenicillin. According to phenotypic analysis, eleven *S. aureus* isolates belonged to MRSA. Since some of them belonged to the same clones (highlighted by color in the table), the actual number of MRSA strains was six. One of them (Crie-F374, ST398) was multidrug-resistant (MDR) as it was also resistant to gentamicin, trimethoprim/sulfamethoxazole, clindamycin, and tetracycline. This isolate was collected in the Vologda region from minced pork cutlet produced by one of the largest manufacturers of semi-finished meat products in Russia. ST398 isolates were also collected earlier from pork meat in Denmark and China [15,23]. The other five MRSA strains in our collection belonged to ST22.

All remaining methicillin-susceptible *S. aureus* (MSSA) isolates differed from each other by having additional resistance to at least one antibiotic—clindamycin or tetracycline. Separate isolates were characterized by additional resistance to linezolid, ciprofloxacin, or cefoxitin (Figure 2). It is worth noting that the latter antimicrobial drug was added to the testing antibiotic panel in 2020.

In silico searching for antimicrobial resistance determinants indicated that MRSA isolates from our collection were characterized by a very limited set of AMR genes (*blaZ* and *mecA)*, except for the only MDR isolate Crie-F374 possessing 11 AMR genes providing resistance to aminoglycosides, beta-lactams, trimethoprim, macrolides, lincosamide, and tetracycline. AMR gene and phenotypic profiles for this isolate were in good correspondence. MRSA isolates belonging to the Dagestan strain (Crie-F275-277) and a sample collected from the Vologda region (Crie-F382) carried the same AMR genes including an additional *inuA* gene determining resistance to lincosamide. Notably, only MRSA isolates possessed *inu* genes in our *S. aureus* collection. The isolates Crie-F377 and Crie-F379 were also positive for *cat* gene (resistance to phenicols) and the former isolate additionally had an *ermC* gene (resistance to macrolides). All MRSA samples under investigation harbored staphylococcal cassette chromosome mec (SCCmec) IVc.

As for the MSSA RTE food isolates, five of them (CrieF-278, 372, 595, 596, and 597) had the only AMR gene—*blaZ*. The maximum number of AMR genes (5) representing the whole spectrum of antimicrobial compounds mentioned above were carried by two isolates Crie-F343 and Crie-F346, respectively, which were collected from distant geographic regions. Their genomic AMR profiles differed from each other by the set of *erm* genes (*erm(A)* and *erm(C)* for CrieF-343 vs. *erm(C)* only for CrieF-346), as well as by *tet* cluster genes and *ant(9)-Ia* revealed in CrieF-343 only. Moreover, the isolate Crie-F343 differed significantly from its relative sample (Crie-F267) by carrying additional *erm(C)* and *tet(K)* genes. These findings may indirectly indicate the plasmid localization of additional AMR genes in the Crie-F343 isolates, which hypothesis was additionally confirmed by BLASTing the contigs containing these genes to the ‘nt’ database (https://www.ncbi.nlm.nih.gov/nucleotide/, accessed on 20 April 2022). Another close isolate Crie-F347 was characterized by 3 AMR genes (*blaZ*, *cat(pC194)* and *tet(K)*). Separate MSSA isolates differed by AMR genes determining resistance to aminoglycosides (*aaC*, *aaD*, and *ant(9)-Ia*), phenicols (*cat* and *fexA*), trimethoprim (*dfrG*), macrolides (genes of *erm* cluster), and tetracycline (genes of *tet* cluster). Interestingly, the ST45 isolate CrieF-274 did not carry any AMR genes. At the same time, only CrieF-528 (MSSA) and CrieF-374 (MRSA) carried the trimethoprim resistance genes (*dfrG* and *dfrK*, respectively), which corresponded with their phenotypes.

### 3.3. Virulence Gene Profiles

The set of virulence factors in *S. aureus* strains is extensive and includes both structural components of the cell and secreted products that play a role in the pathogenesis of infectious diseases [24]. The peculiarity of staphylococci is that a single virulence factor can perform several functions in pathogenesis, as well as multiple virulence factors can perform the same function [25].

We identified the virulence factors in the RTE food *S. aureus* isolates investigated by bioinformatics analysis of WGS data. The complete list of detected virulence genes is shown in Table S3. An extensive spectrum of identified 116 virulence genes included 31 clusters determining genes encoding surface cell-bound proteins of the MSCRAMM family recognizing adhesive matrix molecules (*clfA*, *clfB*, *fnbA*, *emp*, *efb*, *eap/map*, *cna*, *sdrC-E*), the genes whose products are the part of immune evasion mechanisms (*ads*, *coa*, *spa*, *sbi*, *VWbp*), the genes encoding different toxins and extracellular enzymes. The genes involved in the formation of the polysaccharide matrix of biofilms (*icaA-D*, *icaR*), capsule biosynthesis (*cap8*), as well as regulatory proteins, were also identified.

The most common virulence genes found in all isolates under study were *adsA*, *aur*, *cap8*A-G, *cap8*L-P, *esaAB*, *essAB*, *esxA*, *ica*ADR, *isd*, *lip* and *sspBC*. In order to elucidate the presence of distinctive virulence gene patterns, the virulence factors will be discussed for three groups of the isolates: MRSA isolates, isolates with the maximum number of virulence factors, and the remaining MSSA isolates.

Virulence gene patterns of MRSA isolates were very similar (except for several isolates) and quite poor, namely, the isolates carried from 13 to 29 determinants in addition to common virulence genes. No virulence determinants specific to MRSA isolates were found in our *S. aureus* collection. Expectedly, four isolates from the Pskov region belonging to the same ST22 *S. aureus* strain were characterized by identical profiles of virulence determinants. However, one of the three Dagestan isolates of another ST22 MRSA strain (Crie-F277) differed from its two relatives (Crie-F275 and 276) by the presence of *clfB* and *prgl* genes. It was the only sample from our RTE food MRSA collection possessing the latter virulence determinant. These three Dagestan isolates differed from other ST22 MRSA samples due to the absence of *spa* and *tsst1* genes. The isolate Crie-F379 of the same ST draws attention to the presence of the *esxB* gene and seven additional genes of the *esaG* cluster. Both genes encode extracellular proteins important for the establishment of infection in the host. This isolate was characterized by the maximal number of virulence determinants in our MRSA isolates’ subset. MDR MRSA ST398 isolate Crie-F374 collected from the Vologda region differed from other MRSA RTE food samples in our collection by the absence of *chp*, *sak*, and *tsst1* genes and by the presence of *esaG1*, *esaG5*, and *esaG6* (together with Crie-F379) virulence determinants, which represent antitoxin proteins of type VII secretion system.

In silico search for virulence determinants allowed revealing four isolates (Crie-F267, 343, 346, and 347) in our *S. aureus* sample set possessing a slightly broader spectrum of the genes analyzed. The first two ST30 isolates belonged to the same strain and possessed 79 and 80 virulence determinants, respectively. The isolate Crie-F343 differed from its relative by the presence of the *cna* gene.

The isolate Crie-F346 (ST5) was characterized by the greatest number of virulence genes among all samples (86), and it simultaneously carried the genes *sea*, *sec*, and *selk* encoding staphylococcal enterotoxins.

The remaining group of RTE food MSSA isolates included 20 samples and two of them (Crie-F384, 385) belonging to the same strain carried a comparatively low number of virulence determinants and differed from each other by the presence of the *fnbB* gene in the Crie-F384 genome. Three isolates of ST5 (Crie-F271, 375 and 378) possessed very similar virulence gene profiles and were characterized by minor differences in the absence/presence of single genes. For instance, Crie-F271 did not have *chp* and *sea* genes; Crie-F375 was lack of hemolysine-encoding gene *hly* and *sed* gene encoding enterotoxin. Crie-F378 was the only isolate from this group carrying the *tsst1* gene.

Four isolates of ST7 (Crie-F279, 371, 381, and 595) had more differences in the absence or presence of separate genes than those of ST5, namely, *esaG5* and *esaG7*, *fnbA*, *map*, *spa*, and *ssa*. The isolate Crie-F371 was the only carrying *pefB* gene, while all of the ST7 MSSA isolates possessed *lukD* and *sea* genes encoding leuko- and enterotoxins, respectively.

From a food safety concept, *S. aureus* virulence factors encoding toxin production are of special interest since they may be associated with staphylococcal food poisoning [26]. In our *S. aureus* isolates of RTE food origin, all samples except Crie-F375 carried *hly/hla* gene encoding exotoxin alpha-hemolysin, while all isolates had an *hlb* gene of beta-hemolysin.

Leukotoxin gene *lukD* was observed in 14 isolates of our collection regardless of geographic origin and types of RTE food. The enterotoxin gene *sea* that is associated with the severity of infections (sepsis and shock) [27] and promotes bacterial survival in vivo [28] was detected in ten RTE food samples. Different pairs of isolates (Crie-F272, 346; Crie-F271, 378 and Crie-F272, 346) also carried other enterotoxin genes (*sec*, *sed*, and *sel*, respectively). The gene *tsst-1* encoding toxic shock syndrome toxin was observed in 10 isolates, seven of which were ST22 MRSA, including the four isolates from the Pskov region belonging to a single strain. The pathogenic potential of the isolates possessing the genes encoding different types of toxins is notable. For example, apart from the genes of exotoxins, the isolate Crie-F378 carried three enterotoxin genes *sea*, *sed*, and *tsst-1*. The ST5 isolate Crie-F346 from Tumen regions possessed three of the enterotoxin genes mentioned above (*sea*, *sec*, and *sel*).

### 3.4. Plasmid Sequences

The genes providing antibiotic resistance are usually located on mobile genetic elements (MGE) [29]. *S. aureus* contains many types of MGEs, including plasmids, transposons, insertion sequences, bacteriophages, pathogenicity islands, and staphylococcal cassette chromosomes. Plasmids are the major source of dissemination of resistance determinants and virulence factors.

**Figure 3:**
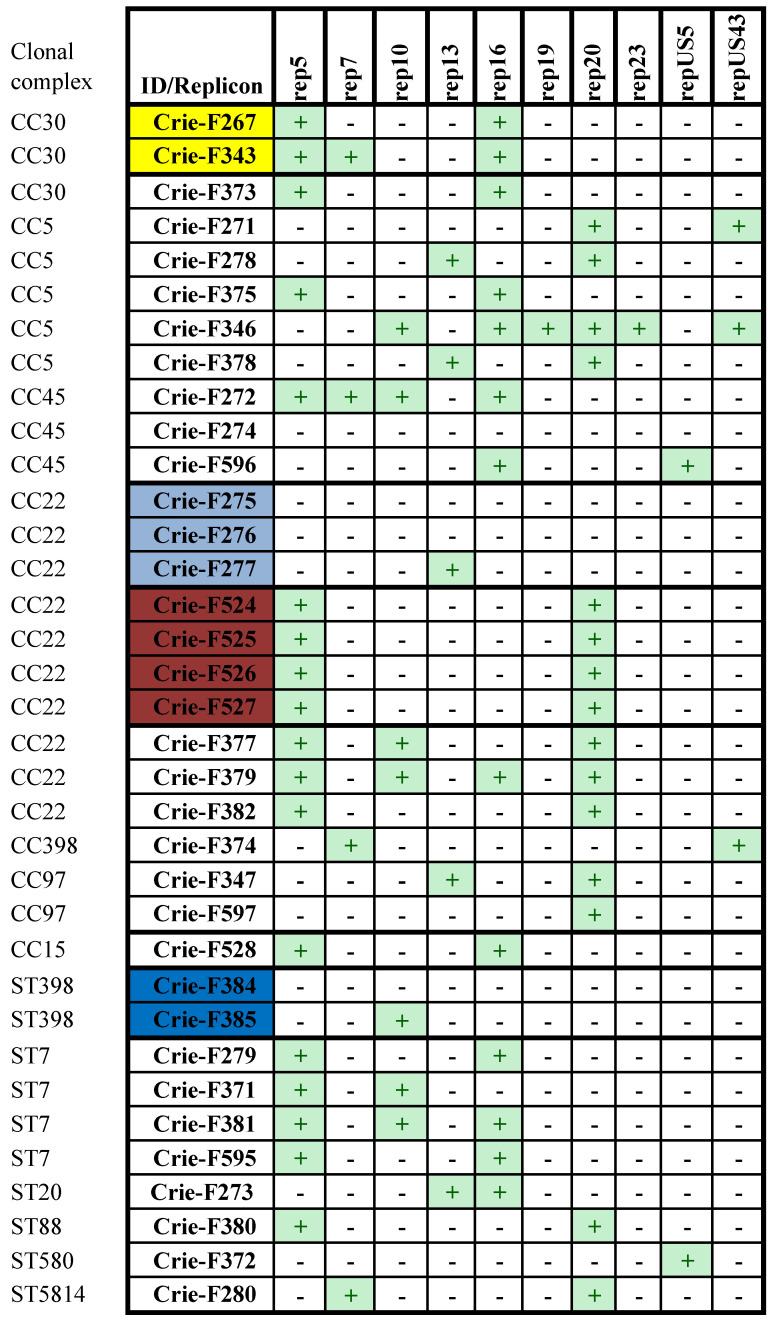
Plasmid replicon sequences profiles of RTE food *S. aureus* isolates studied. ‘+’ means that the given replicon is present in the isolate, ‘-‘ – that the replicon was not found. The isolates found to belong to the same strain are highlighted with color.

The classification of plasmids has been determined by incompatibility groups based on the finding that two plasmids with the same replication (Rep) proteins cannot be stably maintained in the same cell [30]. This method has been developed based on the sequence of the Rep genes [31]. In 35 samples analyzed in the present study, the PlasmidFinder has indicated 69 occurrences of 10 different plasmid replicon sequences (Figure 3).

Four isolates (Crie-F274, 275, 276, and Crie-F384) did not have any Rep-sequences. The isolate Crie-F346 was characterized by the maximal number of Rep-sequences comprising 6 out of 10 types observed. The predominant plasmid replicon sequence found was Rep5 detected in 18 from 35 isolates under investigation. Four MRSA isolates from the Pskov region (Crie-F524-527) belonging to the same strain had the same set of Rep-sequences (Rep5 and Rep20) while two Karelian isolates (Crie-F267 and Crie-F343) presented another epidemiological strain differed by the Rep composition. Apart from the common Rep5 and Rep16, the isolate Crie-F343 carried an additional Rep7 plasmid replicon sequence. Two Dagestan isolates, Crie-F275 and Crie-F276, did not have Rep sequences, while their relative Crie-277 belonging to the same strain possessed Rep13. A similar situation was observed in the case of two Khabarovsk isolates (Crie-F384 and 385) belonging to the same strain. Crie-F385 carried Rep10 only, while Crie-F343 was lack of any plasmid replicon sequences. Two isolates (Crie-F528 and 595) having different STs and collected from different regions and different types of RTE food possessed the same set of Rep sequences (Rep5 and Rep16) representing a major part of the replicons revealed in *S. aureus* isolates studied (18 for Rep5 and 13 for Rep16, respectively).

### 3.5. CRISPR/Cas Systems

Bioinformatics analysis of CRISPR in foodborne pathogens is crucial for assessing their potential evolution in order to predict the outbreaks, which is very important for food safety. At present, the investigations of the CRISPR/Cas systems of pathogenic bacteria of foodborne origin are scarce. In fact, the CRISPR/Cas system in addition to the immune defense function has also been found to be involved in the virulence regulation and formation of biofilms in foodborne pathogens [32].

In our RTE food *S. aureus* collection, three isolates carrying CRISPR/Cas systems of IE type were revealed. Interestingly, the CRISPR/Cas loci of two Karelian samples (Crie-F267 and Crie-F343) belonging to the same strain were significantly different. The Cas cassette of the isolate Crie-F343 consisted of seven genes encoding Cas1 endonuclease, Cas2 integrase, Cas5, Cas6, Cas7, Cse1, and Cse2 proteins. However, the CRISPR/Cas locus of Crie-F267 was deficient and comprised only Cas1 endonuclease and Cas2 integrase. The CRISPR/Cas cassette composition of the third isolate Crie-F346 from the Tumen region was similar to the one of Crie-F343.

Additionally, the genes encoding potential Cas proteins (Cas3_0_I) similar to the CRISPR/Cas type I system were found in the genomes of five isolates (Crie-F271, 278, 375, 378, and 384). The similarity was determined using the CRISPRCasFinder tool [20]. The analysis of these proteins by BLASTp algorithm revealed that Cas3-like proteins of the isolates Crie-F271, 278, 375, and 384 were identical and shared 100% identity with DEAD/DEAH box helicase of *S. aureus*, while Cas3-like protein of the isolate Crie-F378 shared 99% identity with another *S. aureus* DEAD/DEAH box helicase.

## 4. Discussion

The identification of the origin of RTE food-related *S. aureus* strains and evaluation of their pathogenic potential represent very important tasks in the field of food safety investigations. During a one-year period (2019–2020), RTE food samples were collected from cafes and restaurants in different geographic regions of Russia (from Moscow to Kamchatka), corresponding to eight Russian Federal Centers of Hygiene and Epidemiology. In this study, a WGS was used to determine virulence and antimicrobial resistance gene profiles, plasmid sequences, and CRISPR/Cas systems of antibiotic-resistant *S. aureus* isolates collected from various types of RTE foods. To the best of our knowledge, this is the first WGS analysis of this pathogen of RTE food origin in Russia.

The 35 samples analyzed belonged to 15 different MLST-based sequence types and to 20 SpaII types, including four new SSR1 sequences of the SpaII gene, and three groups of AGR system (I-III). The MLST- and Spa-based typing methods provide a high level of standardization for the research and the possibility of effective data exchange between laboratories throughout the world. In addition, Spa-typing allows investigating separate local outbreaks and molecular evolution in general. Notably, 11 out of 35 *S. aureus* isolates under investigation were MRSA isolated from different types of products (bread, salad, meat products, and rolls). The majority of MRSA isolates belonged to CC22 clonal group, while a single MRSA sample (Crie-F374) was characterized by ST398. These results conform well with other studies highlighting that the MSSA population is more heterogeneous than the MRSA population since the former isolates usually constitute a larger number of different clones and lineages [33,34,35].

The MSSA isolates of our sample collection were assigned to CC5, CC30, CC45, CC97, CC398, and other singleton genetic lines. It is well known that strains of CC30 and CC45 are commonly detected in human subjects colonized or infected with *S. aureus* [14]. Moreover, the *S. aureus* CC30 clonal complex has caused a major impact on global human health, triggering three pandemic waves and an epidemic of toxic shock syndrome (TSS). This clonal group is the most common human-colonizing MSSA lineage from which several MRSA clones have emerged [36].

The characterization of phenotypic and genotypic antimicrobial resistance profiles in our RTE-food *S. aureus* isolates revealed a single MDR/MRSA sample (Crie-F374) which was resistant to antimicrobial compounds of five different groups (penicillins, aminoglycosides, trimethoprim/sulfamethoxazole, lincosamide, and tetracycline) and carried a wide repertoire of resistance determinants corresponding to its phenotypic profile. This isolate was collected from a pork cutlet and belonged to CC398 which is associated with food production animals (i.e., LA-MRSA (livestock-associated MRSA)). Our data are in agreement with previous studies highlighting that CC398 MRSA strains usually possess a large variety of resistance genes and show higher levels of multiple drug resistance in comparison to non-CC398 MRSA isolates [37,38]. For instance, CC22 MRSA isolates from our collection were resistant to penicillins only and carried just 2–5 antibiotic resistance determinants (Figure 2). *S. aureus* strains of CC398 were found in livestock around the globe [39]. LA-MRSA strains were also documented in humans proving a zoonotic transmission from animals to humans [5]. Moreover, LA-MRSA prevalence in livestock remains high in many geographical regions, and the acquisition of new virulence and resistance determinants constitutes a growing threat to human health [40]. CC398 MRSA isolates found in humans are usually characterized by two spa-types (t011 and t034) [41], while the isolate Crie-F374 was characterized by a new SSR1 sequence of SpaII gene (Table S1) that has not been found in the respective database (BURP; [42]). Moreover, *sak* and *scn* genes, which are considered to be associated with human infections [14,43,44], were not found in its genome. Thus, one may suppose the livestock origin of this MDR MRSA isolate collected from RTE food proved to be very stable to various “farm-to-fork” conditions.

In our study, all but one (CrieF-274) *S. aureus* isolates from RTE foods were positive for the *blaZ* gene. This rate is very close to the prevalence of *blaZ* among human carriers in Switzerland [14,45] and Germany [46].

The majority of our MRSA isolates carried only a limited number of virulence factors, including the ones facilitating specific adhesion, colonization, invasion, and toxin formation. Such virulence markers as *sak* and *scn* genes were also detected in most isolates studied (29 and 32 samples, respectively), which is likely to indicate their origin from food handlers.

Four samples (Crie-F267, 343, 346, and 347) among our MSSA isolates (*n* = 24) were characterized by a medium variety of AMR determinants (3–5 genes) and a slightly broader spectrum of virulence factors (up to 86 genes). At the same time, these isolates were resistant to two antibiotics only. Interestingly, two Karelian isolates (Crie-F267, 343) representing a single strain differed by AMR gene content. The differences included *erm(c)* and *tet(K)* genes in the genome of the Crie-F343 isolate indicating non-chromosomal localization of these determinants and, therefore, the possibility of their transmission and spreading. The spectra of the virulence determinants of these two isolates were almost the same, except for gene composition in several clusters.

Additionally, three isolates carried putative CRISPR/Cas system of IE-type, which is quite rare for *S. aureus* species [47]. Some pathogens have only a small fraction of the sequenced isolates with CRISPR/Cas, and *S. aureus* is one of them. Only 0.6% (45 of 7865) samples, mainly clinical isolates, had CRISPR/Cas systems [32,47]. For several species it was shown that CRISPR/Cas restricted horizontal gene transfer: *Enterococcus faecalis* [48], *Pseudomonas aeruginosa* [49], and *Acinetobacter baumannii* [50].

In spite of the same system type, the Cas-cassette composition of the isolates representing the same strain differed by a deletion in the case of the Crie-F267 isolate. These isolates also differed by plasmid replicon composition. It is well known that the CRISPR/Cas system has also been found, in addition to the immune defense function, to play the role in hindering the uptake of antibiotic resistance genes, regulating the virulence of bacteria, and influencing the formation of biofilms by foodborne pathogens [32,47,51]. However, three isolates harboring putative CRISPR/Cas systems in our *S. aureus* RTE-food collection carried a broad variety of antibiotic-resistant genes and a rather extensive spectrum of virulence factors. According to the UniProtKB database, Cas3-like proteins of the isolates Crie-F271, 278, 375, and 384 shared homology with DEAD-box ATP-dependent RNA helicase CshA, which played an important role in quorum sensing of *S. aureus* [52]. At the same time, the Cas3-like protein of Crie-F378 was similar to putative ComF operon 1 of *Staphylococcus epidermidis* encoding the purine salvage pathway enzyme. Thus, it could be assumed that the first four isolates possessed additional pathogenic determinants.

We would like to emphasize that the low number of CRISPR/Cas systems in *S. aureus* isolates of different geographic origins may indicate that the presence of such immune systems in this species is a spontaneous biological phenomenon, namely, that such systems could be acquired from some other bacterial species sharing the environment with *S. aureus* [53].

The analysis of plasmid sequences showed that six isolates from our set harbored multiple types (more than 2) of plasmid replicons simultaneously, while only four isolates did not have any Rep-sequences at all. An association between resistance genotype and Rep families, as well as a relationship between separate CC and some Rep sequences, were described for *S. aureus* strains in the previous studies [31,54]. In spite of a small sample set for each CC in our *S. aureus* RTE food collection, the plasmid sequence data obtained are in accordance with the results of the works mentioned above. For instance, the combination of Rep5 and Rep16 families was common for the isolates belonging to CC30 and ST7. At the same time, the presence of Rep5 and Rep20 plasmid replicons was a “signature” of MRSA CC22 isolates.

It is well known that Staphylococci typically carry one or more plasmids per cell and these plasmids include varied gene content [55]. *S. aureus* plasmids can confer resistance to antimicrobials, biocides, and heavy metals [56], and may also encode host survival elements, virulence factors, and toxins [55]. The acquisition of these different genetic elements in a single large plasmid can enhance the adaptation and dissemination of *S. aureus* in different environments due to co-selective advantage [57]. In order to obtain reliable whole-plasmid assemblies, long-read sequencing would be preferable since it provides the necessary resolution to separate genomic and plasmid sequences on the stage of contig assembly [58]. We are planning to sequence the isolates using a MinIon device (Oxford Nanopore Technologies, Oxford, UK) to get deeper insights into plasmid sequences and structure that could facilitate elucidating resistance transfer mechanisms.

At a first glance, the isolates from our collection are mostly non-MDR and do not seem to represent a significant danger to public health. On the other hand, however, given the fact that they were isolated from RTE food, the samples and their sources represent a “means of transfer and dissemination” of important pathogenicity determinants. Moreover, the pathogenic potential of separate isolates studied cannot be ignored, especially for those carrying enterotoxin genes. Our WGS results revealed that 14 samples (40%) carried at least one enterotoxin gene (*sea*, *sec*, *sed*, or *sel*). Additionally, major portion of the isolates harboring the *tsst1* gene were MRSA, and seven out of 10 of them were assigned to CC22 being one of the most important disease-causing clones transmitting rapidly within and between hospitals globally [59]. Therefore, the presence of these pathogenic factors in *S. aureus* isolates from RTE food can pose a significant threat to public health. We can also suggest that such pathogenic potential, including an arsenal of mechanisms providing resistance to antimicrobial drugs, leads to the long-time circulation, transmission, and dissemination of *S. aureus* in the community and, in particular, within the RTE food supply chains. Therefore, the development of monitoring criteria for MRSA and MSSA, as well as for other selected foodborne bacteria, may provide a valuable option for controlling the dissemination of antimicrobial resistance and other pathogenic factors. Such criteria could be based on microbiological properties or antimicrobial resistance potential of the isolates studied.

## 5. Conclusions

This study provides the WGS-based investigation of 35 *S. aureus* from RTE food in various geographic regions of Russia. The isolates were characterized by their phenotypic and genomic AMR profiles, their population structure, the presence of virulence determinants, as well as by plasmid replicon sequences and CRISPR/Cas systems.

To the best of our knowledge, this is the first WGS-based study of such a diverse *S. aureus* population obtained from RTE food in Russia.

We believe that the WGS data obtained will greatly facilitate further studies of foodborne *S. aureus* epidemiology, as well as its genome plasticity, in terms of the acquisition of various genetic elements related to host adaption, antimicrobial resistance, and virulence. In turn, the results of such studies will help to develop preventive measures against human infections caused by this important pathogen in community settings.

## Data Availability

The assembled genome sequences for all isolates were uploaded to the NCBI Genbank under the project number PRJNA823522.

## Supplementary Materials

The following supporting information can be downloaded at: https://www.mdpi.com/article/10.3390/foods11172574/s1, Table S1: Origin and typing of RTE food *S. aureus* isolates studied; Table S2. cgMLST profiles for *S. aureus* isolates from ready-to-eat food; Table S3: Virulence factors revealed in *S. aureus* isolates from ready-to-eat food; Figure S1: Minimum-spanning tree (MST) of cgMLST allelic profiles for *S. aureus* isolates; if a group of the isolates possessed completely the same profiles, only one isolate from this group was shown for clarity.
