# Supplementary material for: Whole-Genome Analysis of Staphylococcus aureus Isolates from Ready-to-Eat Food in Russia
Source: Foods. 2022 Aug 25;11(17):2574. doi: 10.3390/foods11172574 (PMC9455289; doi:10.3390/foods11172574)

Figure S1. Minimum-spanning tree (MST) of cgMLST allelic profiles for *S. aureus* isolates; if a group of the isolates possessed completely the same profiles, only one isolate from this group was shown for clarity

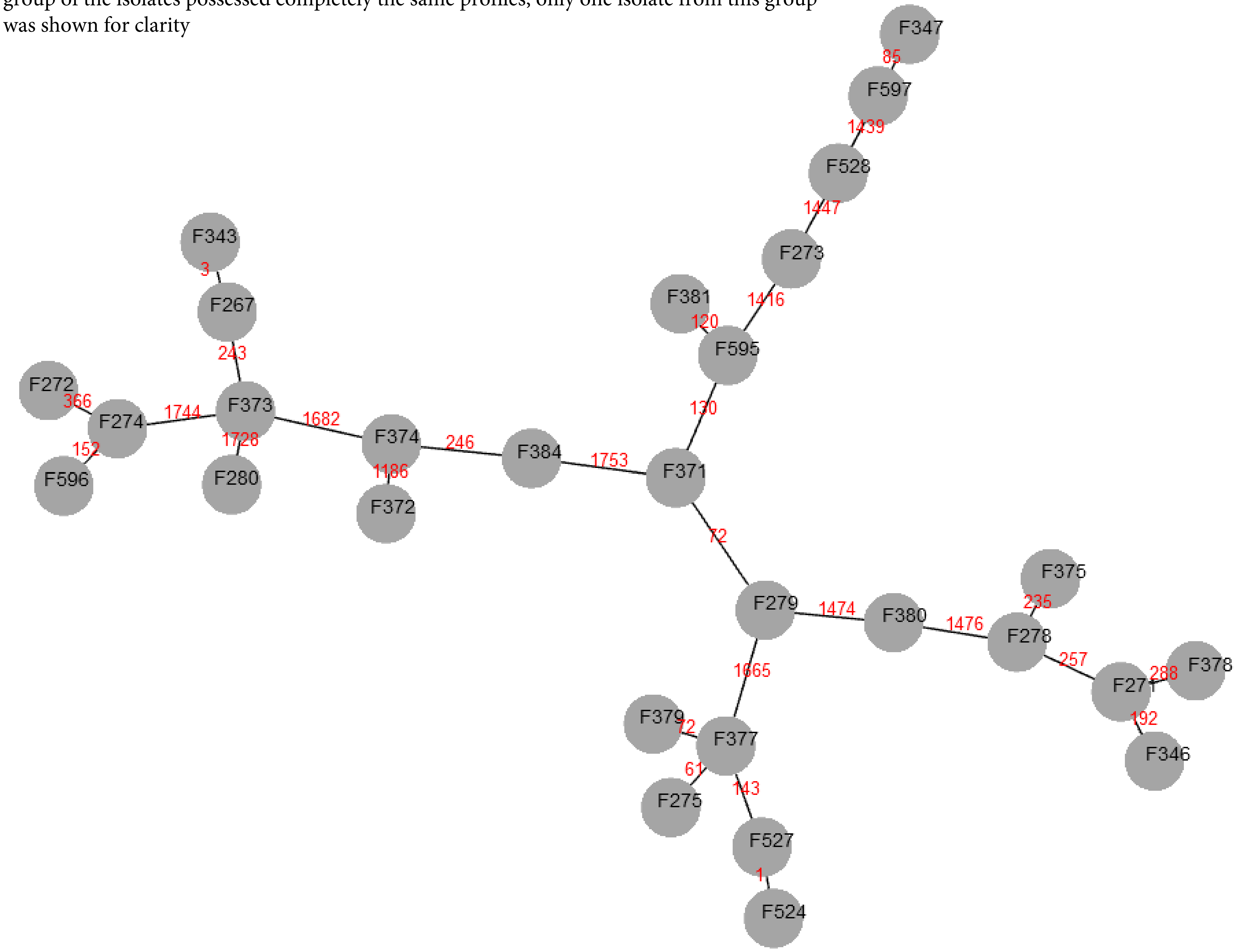

Supplement: Supplementary file 1 [file foods-11-02574-s001.zip › figureS1.pdf]
